# 38 Burn Prevention and Education for Newcomers to North America

**DOI:** 10.1093/jbcr/irad045.012

**Published:** 2023-05-15

**Authors:** Shyla Bharadia, Andrew Galbraith, Alec Lamb, Ali Babwani, Vincent Gabriel, Preet Sahota, Keerthana Chockalingam, Sarthak Sinha

**Affiliations:** University of Calgary, Calgary, Alberta; Canada's Michael Smith Genome Sciences Centre, Vancouver, Alberta; University of Calgary, Calgary, Alberta; University of Calgary, Calgary, Alberta; Calgary Firefighters Burn Treatment Centre, Calgary, Alberta; University of Calgary, Calgary, Alberta; University of Calgary, Calgary, Alberta; University of Calgary, Calgary, Alberta; University of Calgary, Calgary, Alberta; Canada's Michael Smith Genome Sciences Centre, Vancouver, Alberta; University of Calgary, Calgary, Alberta; University of Calgary, Calgary, Alberta; Calgary Firefighters Burn Treatment Centre, Calgary, Alberta; University of Calgary, Calgary, Alberta; University of Calgary, Calgary, Alberta; University of Calgary, Calgary, Alberta; University of Calgary, Calgary, Alberta; Canada's Michael Smith Genome Sciences Centre, Vancouver, Alberta; University of Calgary, Calgary, Alberta; University of Calgary, Calgary, Alberta; Calgary Firefighters Burn Treatment Centre, Calgary, Alberta; University of Calgary, Calgary, Alberta; University of Calgary, Calgary, Alberta; University of Calgary, Calgary, Alberta; University of Calgary, Calgary, Alberta; Canada's Michael Smith Genome Sciences Centre, Vancouver, Alberta; University of Calgary, Calgary, Alberta; University of Calgary, Calgary, Alberta; Calgary Firefighters Burn Treatment Centre, Calgary, Alberta; University of Calgary, Calgary, Alberta; University of Calgary, Calgary, Alberta; University of Calgary, Calgary, Alberta; University of Calgary, Calgary, Alberta; Canada's Michael Smith Genome Sciences Centre, Vancouver, Alberta; University of Calgary, Calgary, Alberta; University of Calgary, Calgary, Alberta; Calgary Firefighters Burn Treatment Centre, Calgary, Alberta; University of Calgary, Calgary, Alberta; University of Calgary, Calgary, Alberta; University of Calgary, Calgary, Alberta; University of Calgary, Calgary, Alberta; Canada's Michael Smith Genome Sciences Centre, Vancouver, Alberta; University of Calgary, Calgary, Alberta; University of Calgary, Calgary, Alberta; Calgary Firefighters Burn Treatment Centre, Calgary, Alberta; University of Calgary, Calgary, Alberta; University of Calgary, Calgary, Alberta; University of Calgary, Calgary, Alberta; University of Calgary, Calgary, Alberta; Canada's Michael Smith Genome Sciences Centre, Vancouver, Alberta; University of Calgary, Calgary, Alberta; University of Calgary, Calgary, Alberta; Calgary Firefighters Burn Treatment Centre, Calgary, Alberta; University of Calgary, Calgary, Alberta; University of Calgary, Calgary, Alberta; University of Calgary, Calgary, Alberta; University of Calgary, Calgary, Alberta; Canada's Michael Smith Genome Sciences Centre, Vancouver, Alberta; University of Calgary, Calgary, Alberta; University of Calgary, Calgary, Alberta; Calgary Firefighters Burn Treatment Centre, Calgary, Alberta; University of Calgary, Calgary, Alberta; University of Calgary, Calgary, Alberta; University of Calgary, Calgary, Alberta

## Abstract

**Introduction:**

The experience of a city’s fire department suggested regions typically home to immigrant and multigenerational families show increased occurrence of fire calls. This study explored the incidence of fire calls in our city stratified by geographic ward and identified opportunities for fire and thermal injury prevention in immigrants.

**Methods:**

This study was approved by our university’s research ethics board. Fire call data from 2016-2021 was used to characterize adjusted fire totals, civilian injuries, incident descriptions, sources of fire ignition, type of material, area of origin, and contribution to ignition of these fires by ward. ANOVAs were performed to reveal differences in fire characteristics between wards. High fire occurrence wards were classed as at greater risk and probable targets for prevention. To follow, an open ended interview was conducted with a newcomer organization in the city to elucidate factors that contribute to thermal injury risk in immigrants. Interview content was informed by a literature search around the factors and impact of burn injury in immigrants to North America, Europe, and Australia. Interview responses were thematically analyzed.

**Results:**

The city is stratified into 14 wards, within which there are several communities described by income, education, immigration diversity and more. Between wards, total adjusted fire incidents were not significantly different (p = 0.119). Though, fire characteristics did differ regionally. Common fire incident descriptions included vehicle/structure, trash and cooking fires with less variability by ward in the number of cooking fires compared to the two former. Contributions to ignition were frequently human related. The supplementary interview highlighted individual burn risk as multifactorial. Cultural norms (notably cooking practices and equipment such as pressure cookers), inexperience (related to weather and frostbite risk), and participation in entry level jobs (including cooking) may impart increased burn risk to immigrants. Senior immigrants were noted to be specifically at risk for burns given their propensity to retain cultural cooking norms.

**Conclusions:**

Newcomers may face increased fire and thermal injury risk that can be attributed to occupational hazards not specific to immigrants and adjustment to a new way of life. Given that many fires and injuries are connected to cooking, kitchen safety and environmental education becomes important to enable safer engagement in cultural practices. Follow up to this work includes matching high fire incidence wards to their resident immigrant groups for targeted prevention.

**Applicability of Research to Practice:**

Inclusion of race and ethnicity in burn registries may assist in recognizing at risk groups for burn prevention and serve as a retrospective comparator to assess the impact of population specific burn prevention efforts.

